# Long Working Hours and Job Quality in Europe: Gender and Welfare State Differences

**DOI:** 10.3390/ijerph15112592

**Published:** 2018-11-20

**Authors:** Lucía Artazcoz, Imma Cortès-Franch, Vicenta Escribà-Agüir, María López, Fernando G. Benavides

**Affiliations:** 1 Agència de Salut Pública de Barcelona, 08023 Barcelona, Spain; icortes@aspb.cat; 2 CIBER en Epidemiología y Salud Pública (CIBERESP), 28029 Madrid, Spain; fernando.benavides@upf.edu; 3 Center for Research in Occupational Health, Universitat Pompeu Fabra, 08002 Barcelona, Spain; maria.lopez@upf.edu; 4 Institute of Biomedical Research (IIB-Sant Pau), 08041 Barcelona, Spain; 5 Departament de Pediatria, Obstetrícia i Ginecologia, i de Medicina Preventiva, Facultat de Medicina, Universitat Autònoma de Barcelona, 08193 Barcelona, Spain; 6 Centre for Public Health Research (Health Inequalities Area), Nursing Department, University of Valencia, 46010 Valencia, Spain; escriba_vic@gva.es; 7 Valencian School for Health Studies, Regional Ministry of Health. Generalitat Valenciana, 46010 Valencia, Spain

**Keywords:** gender, working time, socioeconomic factors, working conditions

## Abstract

Chronic extreme long working hours (LWH) have been found consistently associated with poor health status. However, the evidence for moderately LWH (41–60 h a week) is contradictory. Although poor job quality has been proposed as one of the mechanisms of this relationship, there are almost no studies about LWH and job quality. The objectives of this study were to analyze the association between moderately LWH and job quality in the EU27, as well as to examine differences by welfare regimes and gender. This is a cross-sectional study based on data from the 2010 European Working Conditions Survey. A subsample of employees from the EU27 aged 16–64 years who worked 30–60 h a week was selected (12,574 men and 8787 women). Overall, moderately LWH were not consistently associated with poor job quality except among women from Eastern European countries. Therefore, in the EU27 poor job quality does not seem to explain the relationship between moderately LWH and poor health status. The findings among women from Eastern European countries may be related to their weakened position in the labor market and to their work-family conflict resulting from a process of re-familisation that constrains their choices for a good job.

## 1. Introduction

In recent years the number of studies about the relationship between long working hours (LWH) and health has increased. However, findings are still contradictory [1]. A recent systematic review reported associations between LWH (defined as working over 40 h a week) and a range of health outcomes. Yet, although most studies found consistent associations with working ≥55 h a week, results for moderately LWH were not consistent [2]. Chronic extreme LWH (>60 h a week), which are quite common in Asia [3], are likely to lead sooner or later to health problems, even in case of a well-designed psychosocial work environment, because they require constant effort of employees and prohibits adequate recovery [4]. However, very LWH are not common in Europe—mainly among women—where the European Working Time Directive [5] sets a maximum of 48 h per week. The association between moderately LWH and health does not seem to be simple and straightforward, but to depend on some other factors [1,6]. Although poor job quality has been proposed as one of the mechanisms of this relationship [4,7], there are almost no studies about LWH and job quality.

The moderating effect of the organizational context on the relationship between LWH and health outcomes has been identified as one of the critical research needs in the field of LWH [8]. For example, a study carried out in the US reported that variables related to LWH included working more than one job, working split/irregular/on-call shifts and mandatory overtime [9]. Additionally, mandatory overtime has been related to difficulty finding alternative jobs, low say about the job and poor relationships with management [10]. It has been reported that even a limited number of hours of involuntary overtime is associated with poor mental health, but only in low reward situations [11]. Therefore, moderately LWH could be more risky if they are concentrated in poor quality jobs [12,13] since time spent in poor quality jobs is unlikely to be comparable in terms of their health impact to that spent in more rewarding professional activities [4,7,14].

Some studies carried out in the European Union have observed gender and welfare state differences in the relationship between moderately LWH and health status [15,16]. For example, a study based on the 2010 European Working Conditions Survey (EWCS) found that in the EU27 working moderately long hours (≤60 h a week) was consistently associated with poor health status and poor psychological wellbeing in both sexes in Anglo-Saxon countries and primarily among women in Continental and Southern European countries. No associations were observed in Nordic and Eastern European countries [15]. The reasons for these differences between welfare regimes are not well understood. If the relationship between LWH and health status was mediated by poor job quality, we can expect job quality to be poorer among the groups where LWH were related to poor health outcomes.

### 1.1. Job Quality

Job quality has been a key objective of the European Unión in the last decade. A worker’s job quality has profound consequences for his or her psychological, social and economic well-being [17]. A link has also been shown to exist between job quality and productivity [18]. Moreover, job quality affects retirement decisions, and is a key factor contributing to prolong people’s working lives, an important policy objective in ageing societies [19].

There is no universally-accepted definition of job quality but it can be defined as the extent to which a job has work and employment-related factors that foster beneficial outcomes for the employee, particularly psychological and physical well-being, as well as job satisfaction [20]. It is generally agreed that job quality is a multidimensional phenomenon. Besides the difficulties related to the weighting of job quality indicators, the use of synthetic indicators could mask different job quality typologies. Therefore, in this study we examined the different dimensions and sub-dimensions of job quality separately to better understand the diverse nature of LWH across gender and welfare regimes. We measured job quality through an adaptation of the job quality framework developed by Green and Mostafa [20], based on the EWCS, which has four dimensions: intrinsic job quality, prospects, rewards and working time quality. Since job quality is associated with job satisfaction, psychological and physical well-being, closely related to job stress, for the three first dimensions, we selected those factors showing the best fit with the two theoretical models that have been particularly successful in generating and guiding job stress research: the job demand-control-support model and the model of effort-reward imbalance [21]. Nevertheless, none of these models includes working time quality. As working time is one of the main dimensions of gender inequalities [22], and job quality is poorer among women [23], working time quality is a key dimension in examining job quality and LWH from a gender perspective.

There is strong evidence of cross-national variation in job quality as a result of differences in national institutional regimes mainly related to labor market structures and workers’ power [24,25,26]. Moreover, differences in welfare regimes have been reported to result in cross-national variation, not only in the level, but also in the nature of job quality [27].

### 1.2. Long Working Hours

Family responsibilities and financial strain clearly influence the number of hours worked. Workers who want more time with family may prefer fewer hours, while those in economic difficulties may prefer more hours [8].

Several studies have emphasized the role of choice in the relationship between LWH and health status [28,29]. For example, many workers, primarily breadwinners, are obliged to accept LWH simply to service their family debt [30,31]. Additionally, bargaining power considerations suggest that where workers are in situations of vulnerability (such as those working in non-unionized workplaces, on temporary contracts, receiving low wages, or with difficulties of employability) they are more likely to be forced to work long hours [32]. This situation of vulnerability could be associated with the acceptance of poor employment and working conditions.

However, individuals’ work hours are motivated, not only by their own individual circumstances, but by the cultural, institutional, and regulatory environments of the society, which are closely related to welfare state regimes [33]. Regarding labor markets regulation, collective agreements play a dominant role in determining working hours, while statutory maximum hours provide a safeguard for unorganized workers. On the other hand, given the temporal constraints that family responsibilities typically impose on women’s participation in paid employment, the average actual hours of work of employed men exceed those of employed women. Additionally, the traditional role of men as family breadwinners is more likely to be related to forced LWH when family financial stress exists.

### 1.3. Welfare State Regimes

In Europe, there are large differences in the prevalence of LWH among different welfare regimes, which are related to a great extent to gender, labor and family policies. Although seminal research on welfare state typology did not focus on the relationship between work and family policy [34], there is an institutional compatibility of both policies [35]. A family policy model is anchored in a particular welfare regime, that is, in a distinctive allocation of roles in welfare provision among the family, the state, and the market. Actually, Esping-Andersen [36] argues that family policy regimes essentially overlap with his classification of welfare states: Nordic family policy regime (social democratic welfare state), Continental (conservative welfare state), and the Anglo-Saxon family policy regime (liberal welfare state). These welfare regimes are also closely related to working time policies [22]. Two additional types of welfare regimes have been identified: the Southern welfare regime [37] and the post-socialist welfare regime [38].

The Nordic countries are characterized by dual earner/dual carer family models, extensive statutory support for work–family reconciliation, and regulated labor markets. Work-time practices also contribute to achieving a balance between work and family since the proportion of employees working more than 40 h a week is limited. Continental countries have strong labor market regulations, male breadwinner family models, primary welfare responsibilities lie with the family and work-family balance policies are relatively undeveloped. Men usually work a 40-h work week and institutional barriers have limited longer hours [22]. Anglo-Saxon countries are characterized by a strong breadwinner model, part-time employment is concentrated in female-dominated sectors, while flexibility in male jobs entails LWH. Formal childcare is generally provided by “marketized” services. In the UK there is an individual “opt out clause” that permits individual employees to voluntarily work hours more than the 48-h a week. However, it has been suggested that workers may “opt” to work beyond this threshold forced in different ways due to the need to earn more money or pressure exerted by the company for which they work [39]. Southern European countries are characterized by strong labor market regulations with a long work week and low labor force participation rates among married women. In these countries, since the onset of the economic crisis, deregulation began to affect both permanent and temporary workers and precariousness affects the entire workforce [37]. Eastern European countries have a tradition of LWH, labor markets mix characteristics of Anglo-Saxon and Nordic countries, and combine dual earner families with a traditional division of housework and social care for children aged three to six years [38]. In these countries, both standard and non-standard forms of employment are exposed to precarious conditions following various degrees of labor market deregulation. Additionally, the post-1989 economic transformation was accompanied by policies that undermine women’s employment and shift care work back to families. Now the majority of the countries encourage women to leave the labor market to raise children through long parental and care-leaves mostly until the child is 3 years old [40].

The objectives of this study were: (1) to analyze the relationship between moderately LWH and job quality in the EU27, and (2) to examine the potential different patterns by welfare state typologies and gender. We hypothesize that, if the relationship between moderately LWH and poor health status is mediated by poor job quality, LWH is related to overall poor job quality in both sexes in Anglo-Saxon countries and primarily among women in Continental and Anglo-Saxon countries. No or minor associations are expected among workers from Nordic and Eastern European countries.

## 2. Methods

### 2.1. Data

Cross-sectional study based on data from the 5th EWCS of 2010, the survey covering by far the widest range of areas of work and employment quality. Details of the survey are reported elsewhere [41]. For the purposes of this study a subsample of all employees from the EU27 aged 16–64 years who worked 30–60 h a week and had only one job was selected. In order to avoid the effect of very LWH, people working more than 60 h a week were excluded (1.0%). The final sample under analysis was composed of 12,574 men and 8787 women.

### 2.2. Variables

The main exposure variable was the average number of hours worked in a person’s main job per week (94.2% of people in the sample held only one job). This was split into three groups (30–40 h, 41–50 h and 51–60 h), with 30–40 h used as the reference category. The measurement of job quality was based on four dimensions [20]: intrinsic job quality (time pressure, physical demands, skill discretion, autonomy, participation and support), job prospects (type of contract and employer-funded training), rewards and working time quality (changing schedules and work-life conflict).

Regarding intrinsic job quality, the time pressure variable was the mean of two factors, working at very high speed and working to tight deadlines, both on a 7-point scale (Cronbach’s alpha = 0.75). Physical demands were constructed with the mean of four factors, tiring or painful positions, carrying or moving heavy loads, standing and repetitive hand or arm movements on a 7-point scale (Cronbach’s alpha = 0.71). A similar process was followed to construct the skill discretion variable from four questions, assessing the quality of their own work, solving unforeseen problems, doing complex tasks and learning new things, on a 2-point scale (Cronbach’s alpha = 0.71). Autonomy was measured with three questions about the possibility of choosing or changing the order of tasks, the methods of work and the speed or rate of work, on a 2-point scale (Cronbach’s alpha = 0.78). The level of participation in decisions was assessed with three questions about whether people are consulted before targets for their work are set, whether they are involved in improving the work organization or work processes of their department and whether they can influence decisions that are important for their work, on a 5-point scale (Cronbach’s alpha = 0.77). Support was the mean of two questions about the support from colleagues and the manager at work, on a 5-point scale (Cronbach’s alpha = 0.68).

Regarding job prospects, type of contract had five categories: Permanent, fixed-term temporary, temporary employment agency, no contract and other. For the multivariate analysis this variable was dichotomized by combining the latter four categories. Employer-funded training was measured through two questions about training paid for or provided by the employer and on-the-job training, on a 2-point scale (Cronbach’s alpha = 0.61).

Rewards were appraised through workers’ responses to four questions about pay, career advancement, feeling at home in the organization and motivation, on a 5-point scale (Cronbach’s alpha = 0.71).

Regarding working time quality, changing schedules was assessed with the questions “Do changes to your work schedule occur regularly? (if yes) How long before are you informed about these changes?” A dichotomic variable was created with categories “Yes, the same day”, “Yes, the day before” and “Yes, several days in advance”, being considered as changing schedules. Work-life conflict was assessed with a question about whether the working hours fit in with the family or social commitments outside work. The variable was dichotomized.

Countries were grouped into five widely used welfare state typologies including all countries of the EU27 [42]: Continental (Germany, France, Austria, Belgium, the Netherlands and Luxembourg), Anglo-Saxon (the UK and Ireland), Eastern European (Latvia, Poland, Rumania, Bulgaria, Czech Republic, Estonia, Hungary, Lithuania, Slovenia and Slovakia), Southern European (Spain, Italy, Cyprus, Greece, Malta and Portugal) and Nordic countries (Finland, Denmark and Sweden).

The models were adjusted for age (measured continuously) and occupational category. Occupational category was measured through the respondent’s current occupation based on the 2008 International Standard Classification of Occupations one-digit categories and grouped into three categories: upper (1 and 2), medium (3 to 5) and lower (6 to 9).

### 2.3. Statistical Analysis

First, gender differences by country typologies for all the dependent and independent variables were tested at the bivariate level using the chi-square test for categorical variables, the *t*-test for age and the Kolmogorov-Smirnov test for job quality variables that were measured through scales. Second, multiple logistic regression models adjusted for age and occupational category separated by gender and country typologies were fitted in order to test the association of working time with each job quality indicator. For the multivariate analysis, all the scales were dichotomized about the median of each gender and country typology. Trends were assessed with the Wald test. All analyses included the weights derived from the complex sample design.

## 3. Results

In all country typologies men were more likely to work long hours. The prevalence of LWH was higher in Anglo-Saxon and Eastern European countries. Job quality indicators differed by gender and welfare state typologies. In general, the quality of employment was higher in the Nordic countries where, moreover, no gender differences were observed (Table 1).

Table 2 shows the association between working hours and job quality among men. We observed four systematic associations involving time pressure, non-permanent contracts, changing schedules and work-life conflict. In all country typologies long hours were related to lower time pressure. Conversely, in all welfare state typologies, except Nordic countries, LWH were associated with changing schedules, with a significant gradient, as well as to work-life conflict and a trend was also found. Moreover, in all countries LWH were positively related to non-permanent contracts, although differences were statistically significant only for Anglo-Saxon, Eastern and Southern European countries. In Nordic countries, only a positive association between working 51–60 h a week and work-life conflict was observed.

The results for women are presented in Table 3. As among men, women working long hours were more likely to work on non-permanent contracts, although differences were statistically significant only for Southern and Eastern European countries, and to have work-life conflict, whereas they were less likely to report time pressure. However, an association with changing schedules was only observed in Anglo-Saxon and, to a lesser extent, in Eastern European countries. A systematic association with poor job quality indicators—except time pressure and rewards—was observed among women from Eastern European countries. Conversely, no associations were observed among women from Nordic countries.

## 4. Discussion

As has been mentioned before, a previous study also based on the 2010 EWCS, found that moderately LWH were related to poor health outcomes in both sexes in Anglo-Saxon countries, and primarily among women in Continental and Southern European countries. No associations were observed in Nordic and Eastern European countries [15]. However, contrary to what we expected, the patterns of association between LWH and job quality were different since we only found a consistent association between LWH and poor job quality among women from Eastern European countries. Therefore, our results do not support the hypothesis that poor job quality mediates the relationship between moderately LWH and health status.

This study found other relevant results regarding the job quality of LWH in the EU27: (1) overall, LWH were not consistently associated with poor job quality; (2) in both sexes LWH were positively associated with non-permanent contracts and work-life conflict but with less time pressure; (3) among men, LWH were consistently related to changing schedules; and (4) only among women from Eastern European countries a consistent positive association between LWH and most poor job quality indicators was found.

### 4.1. General Patterns

In Nordic countries, and in both sexes, there were almost no associations between LWH and job quality, whereas in the rest of country typologies, except among women from Eastern European countries, about the same number of positive and negative associations were observed, although no consistent patterns were observed. These results are in line with previous studies that have reported that the relationship between LWH and job psychosocial factors differs depending on the factor being examined [9].

There was a consistent negative correlation between LWH and time pressure. This finding suggests that LWH are related to less work intensity. Conversely, in both sexes LWH were related to non-permanent contracts, although differences were only statistically significant among men in Anglo-Saxon countries, characterized by deregulated labor markets, and in both sexes in Eastern and Southern European countries, with a high level of precariousness of the whole labor market. These findings suggest a forced nature of LWH for many workers who are in a position of low bargaining power due to their job instability. Additionally, there was a consistent positive association with work-life conflict, which is in line with studies about work-family conflict [43,44].

Unlike women, among men LWH were consistently related to changing schedules, suggesting an association with overtime. During the economic crisis, in Europe overtime has been regarded by many employers as a vital element in achieving flexibility and, on the other hand, by many employees as an important source of income [45]. Although family models differ by welfare regimes, in all countries men are more likely than women to be the main contributors to household incomes which is consistent with overtime being more frequent among them [46]. However, it remains a very irregular source of income for employees, as they can work overtime according to the needs of the business and to fluctuations in demand and cannot therefore rely on it as regular and foreseeable income. Moreover, it has been reported that schedule variability can affect the ability to plan for sleep and recuperation, and to arrange for child care and other family responsibilities.

### 4.2. LWH and Poor Job Quality among Women from Eastern Europe

We only found a consistent association between LWH and almost all poor job quality indicators among women from Eastern European countries. These countries have a tradition of LWH and overtime payments often constitute a regular and substantial element of wage packages and are relied on to ensure a decent standard of living [47]. Labor markets attributes high value to employees who are flexible in working hours, workplace and type of contract, and who are willing to work long hours. However, they typically penalize disadvantaged employees with caring responsibilities, who are usually women. Labor market policies make it difficult for women to return to work after having been on parental leave for several years. Although there is a legal guarantee to return to one’s job after parental leave, these countries have not enforced it. This contributes to a widespread feeling of insecurity among women, as employers often lay off mothers returning from their parental leave. Moreover, post-communist women believe that they must and should work to support their families [48]. As a result, precarious work has acquired a specific form for women who are forced to accept precarious jobs when they have care responsibilities as a temporary strategy that may turn into a trap excluding them from a better job [40,49]. According to our results, precariousness is strongly correlated with LWH among women from Eastern European countries. Given the high proportion of women working long hours in these countries, these results are particularly relevant.

### 4.3. Strengths and Limitations

As far as we know this is the first study about moderately LWH and job quality carried out in a large and representative sample of the EU27 and examining a broad range of job quality indicators. Although the cross-sectional design represents a study limitation, a major strength is the large sample size and the rigorous quality-protocol of the EWCS [42]. Although this study is based on data from 2010, in our opinion the results are still applicable because no significant changes have occurred during this period regarding LWH in Europe. There is no particular welfare state classification of welfare state regimes that has been accepted as the standard, but that used in this study is one of the most empirically accurate [50] and is consistent with the hypothesis about country-level factors related to LWH. Although there clearly is variability between countries within each welfare regime, it is probably much lower than the differences between country typologies.

## 5. Conclusions

Contrary to what we hypothesized, the patterns of association between LWH and job quality were not consistent with those between LWH and health status. Therefore, in the EU27 poor job quality does not seem to explain the relationship between moderately LWH and poor health status. LWH were only consistently associated with poor job quality among women from Eastern European countries. This finding may be related to their weakened position in the labor market and to their work-family conflict resulting from a process of re-familisation that constrains their choices for a good job. In both sexes LWH were positively associated with non-permanent contracts and work-life conflict but with less time pressure. Among men, they were also related to changing schedules.

These results remark the need for more research about LWH and job quality examining separately the different job quality indicators in order to better understand the nature of job quality of LWH across welfare regimes and gender categories. Moreover, the poor job quality of LWH among women from Eastern European countries deserves further attention.

## Figures and Tables

**Table 1:** General description of the population by sex and country group. Employees working 30–60 h a week. (%). 5th European Working Conditions Survey, 2010.

	Continental		Anglo-Saxon		Eastern European		Southern European		Nordic	
	Men	Women	Men	Women	Men	Women	Men	Women	Men	Women
	*N* = 4887	*N* = 3298	*N* = 1718	*N* = 1003	*N* = 2267	*N* = 2042	*N* = 3189	*N* = 1963	*N* = 514	*N* = 481
Paid working hours (%)										
30–40 h	78.3 ^c^	87.8	63.6 ^c^	80.6	65.4 ^c^	74.5	75.4 ^b^	79.9	79.6	84.0
41–50 h	18.4	11.0	26.3	16.8	26.2	21.2	20.7	16.8	17.8	14.1
51–60 h	3.3	1.2	10.1	2.6	8.4	4.4	3.9	3.3	2.7	1.9
Occupational category (%)										
Upper	20.8 ^c^	23.1	36.7 ^c^	38.7	14.3 ^c^	19.8	11.7 ^c^	18.2	23.5 ^c^	33.4
Middle	33.8	64.9	28.8	50.8	26.1	55.1	34.8	61.9	36.8	54.7
Lower	45.4	12.0	34.4	10.4	59.6	25.1	53.5	19.8	39.7	11.9
Intrinsic job quality (mean; SD)										
Time pressure	4.0 (1.7) ^c^	3.7 (1.8)	3.8 (1.8)	3.8 (1.9)	3.5 (1.8) ^c^	3.3 (1.9)	3.7 (1.8) ^c^	3.3 (1.8)	3.8 (1.5)	4.0 (1.7)
Physical demands	3.3 (1.5)	3.2 (1.5)	3.2 (1.5)	2.8 (1.3)	3.4 (1.6) ^c^	3.0 (1.5)	3.7 (1.6)	3.3 (1.4)	3.2 (1.4)	3.1 (1.4)
Skill discretion	0.8 (0.3)	0.7 (0.3)	0.8 (0.3)	0.8 (0.3)	0.7 (0.3)	0.7 (0.3)	0.7 (0.3) ^c^	0.6 (0.3)	0.9 (0.2)	0.8 (0.2)
Autonomy	0.6 (0.4) ^a^	0.7 (0.3)	0.7 (0.4) ^a^	0.7 (0.4)	0.5 (0.4)	0.5 (0.4)	0.6 (0.4)	0.6 (0.4)	0.8 (0.3)	0.8 (0.3)
Participation	3.0 (1.1) ^b^	2.9 (1:1)	3.3 (1.3)	3.3 (1.1)	3.0 (1.1) ^b^	2.9 (1.1)	2.8 (1.2) ^b^	2.7 (1.2)	3.5 (1.0)	3.5 (0.9)
Support	3.6 (1.0)	3.7 (1.0)	4.1 (0.9) ^a^	4.2 (0.8)	3.9 (0.9)	3.8 (1.0)	3.8 (1.0)	3.8 (1.0)	4.0 (0.8)	4.0 (0.9)
Prospects										
Non-permanent contract (%)	11.1 ^c^	11.9	15.1 ^b^	12.9	15.0 ^b^	16.3	10.7 ^c^	14.6	9.6	10.7
Employer-funded training (mean; SD)	0.4 (0.4)	0.4 (0.4)	0.5 (0.4)	0.5 (0.4)	0.3 (0.4)	0.3 (0.4)	0.3 (0.4)	0.3 (0.4)	0.6 (0.4)	0.6 (0.4)
Rewards (%)	2.6 (0.8) ^c^	2.7 (0.8)	2.5 (0.8) ^c^	2.4 (0.8)	2.8 (0.8) ^a^	2.9 (0.8)	2.8 (0.8) ^a^	2.9 (0.7)	2.5 (0.7)	2.5 (0.7)
Working time quality										
Changing schedules	27.6	25.9	23.8 ^c^	17.1	27.3 ^c^	19.3	21.0 ^b^	16.9	12.7	15.2
Work-life conflict	18.8	19.0	17.1 ^c^	11.2	19.0 ^b^	15.5	26.2	24.8	12.0	11.3
Age (mean; SD)	41.3 (10.9) ^c^	40.1 (10.9)	39.7 (11.7) ^a^	38.5 (11.6)	39.3 (11.1)	39.7(10.4)	40.5 (10.7) ^a^	39.8 (10.2)	42.4 (10.9)	43.5 (11.2)

^a^*p* < 0.05. ^b^ *p* < 0.01.^c^ *p* < 0.001. *p*-values compare men and women in each country typology.

**Table 2:** Association of working time with poor job quality by country group among men. Adjusted odds ratios (aOR) and 95% confidence intervals (95% CI). Employees working 30–60 h a week. 5th European Working Conditions Survey, 2010.

	Continental			Anglo-Saxon			Eastern European			Southern European			Nordic		
	*N* = 4887			*N* = 1718			*N* = 2267			*N* = 3189			*N* = 514		
	%	aOR	95%CI	%	aOR	95%CI	%	aOR	95%CI	%	aOR	95%CI	%	aOR	95%CI
**Intrinsic job quality**															
High time pressure															
30–40 h	37.9	1 ^f^		42.5	1 ^d^		57.2	1 ^f^		48.2	1 ^f^		46.2	1 ^e^	
41–50 h	31.6	0.7	0.59–0.82 ^c^	42.5	1.06	0.85–1.35	38.6	0.46	0.38–0.56 ^c^	31.9	0.51	0.43–0.63 ^c^	27	0.38	0.22–0.65 ^c^
51–60 h	27.5	0.53	0.36–0.76 ^b^	28.4	0.56	0.37–0.75 ^b^	33.3	0.41	0.29–0.56 ^c^	27.3	0.37	0.25–0.59 ^c^	35.7	0.44	0.14–1.44
High physical demands															
30–40 h	51.2	1		57	1		50.1	1 ^d^		52.3	1		57.4	1	
41–50 h	41.5	0.86	0.72–1.02	44.4	0.48	0.37–0.63 ^c^	52.4	1.14	0.92–1.41	54.3	1.17	0.96–1.42	42.7	0.79	0.46–1.36
51–60 h	43.5	1.01	0.70–1.47	59.8	1.7	1.17–2.46 ^b^	62.2	1.43	1.01–2.03 ^a^	50	1.06	0.64–1.64	30.8	0.82	0.22–3.05
Low skill discretion															
30–40 h	31.6	1 ^f^		46.1	1		41	1 ^e^		37.9	1 ^e^		42.2	1	
41–50 h	24.2	0.82	0.69–0.98 ^a^	43.2	0.9	0.71–1.15	36.9	0.88	0.66–0.99 ^a^	47.5	1.53	1.27–1.84 ^c^	28.1	0.59	0.35–1.01
51–60 h	13.7	0.4	0.25–0.64 ^c^	32	0.7	0.49–1.01	34.4	0.68	0.49–0.95 ^a^	27.3	0.72	0.46–1.11	28.6	0.77	0.22–2.71
Low autonomy															
30–40 h	45.4	1		50.8	1		51.3	1		45.9	1		38.9	1	
41–50 h	39	1.02	0.85–1.22	49.4	1.03	0.80–1.32	48.7	0.88	0.71–1.10	42	0.83	0.68–1.02	37.1	1.2	0.71–2.02
51–60 h	36.6	0.81	0.55–1.18	41.8	1.06	0.74–1.52	47.5	0.77	0.55–1.09	41.2	0.95	0.62–1.46	28.6	1.13	0.31–4.16
Low participation															
30–40 h	43.4	1 ^f^		49.1	1 ^d^		45	1 ^e^		50.1	1		53.1	1	
41–50 h	29	0.64	0.54–0.76 ^c^	39.8	0.74	0.56–0.97 ^a^	39.9	0.78	0.64–0.96 ^a^	52.1	1.12	0.93–1.34	44.2	0.98	0.57–1.68
51–60 h	34.8	0.82	0.58–1.17	36.1	0.79	0.53–1.17	41.5	0.75	0.54–1.03	40	0.82	0.55–1.23	41.7	1.2	0.35–4.12
Low support															
30–40 h	49.6	1		31.8	1		39.8	1		40.3	1 ^f^		33.9	1	
41–50 h	49.8	1.04	0.89–1.21	28.4	0.89	0.69–1.54	42.9	1.13	0.93–1.37	45.6	1.24	1.02–1.48 ^a^	35.2	1.01	0.61–1.69
51–60 h	41.3	0.74	0.53–1.02	29.1	0.96	0.66–1.39	44.9	1.19	0.87–1.63	50	1.71	1.17--2.49 ^b^	46.2	1.39	0.45–4.30
**Prospects**															
Non-permanent contract															
30–40 h	10.1	1		14.6	1		14	1		18.5	1 ^f^		9	1	
41–50 h	9.7	1.04	0.81–1.34	13.5	0.89	0.64–1.24	17.7	1.33	1.03–1.73 ^a^	27.6	1.51	1.22–1.86 ^c^	12.4	1.22	0.56–2.65
51–60 h	10.6	1.25	0.74–2.12	21.9	1.73	1.14–2.64 ^a^	14.4	0.98	0.64–1.53	27.3	1.45	0.93–2.27	14.3	1.89	0.36–9.50
Low employer-funded training															
30–40 h	50.3	1 ^e^		37.5	1		51.2	1		61.4	1		30.9	1	
41–50 h	41.6	0.8	0.69–0.94 ^b^	33.3	0.79	0.62–1.00	55.7	1.2	0.98–1.46	58	0.9	0.76–1.09	30.7	1.26	0.75–2.12
51–60 h	40.4	0.71	0.51–1.00 ^a^	30.2	0.77	0.54–1.11	54.3	1	0.73–1.37	53.7	0.92	0.62–1.35	15.4	0.55	0.13–2.43
**Rewards**															
30–40 h	40.6	1		46.5	1 ^e^		42.9	1		46.2	1		45.2	1	
41–50 h	42.3	0.99	0.85–1.15	58.3	1.55	1.22–1.95 ^c^	41.7	0.97	0.80–1.19	40.1	0.76	0.63–0.91 ^b^	56.2	1.25	0.77–2.02
51–60 h	48.4	1.22	0.88–1.69	55.6	1.24	0.88–1.75	40.6	0.95	0.69–1.31	52.5	1.17	0.80–1.71	61.5	1.49	0.47–4.70
**Working-time quality**															
Changing schedules															
30–40 h	26.1	1 ^f^		21.2	1 ^f^		22.6	1 ^f^		19.1	1 ^f^		14.1	1	
41–50 h	33.8	1.65	1.40–1.97 ^c^	25.9	1.42	1.06–1.82 ^a^	33.4	1.72	1.39–2.13 ^c^	25.9	1.41	1.15–1.74 ^b^	5.7	0.49	0.19–1.23
51–60 h	29.8	1.46	1.02–2.08 ^a^	34.7	2.48	1.71–3.59 ^c^	45.4	2.79	2.03–3.85 ^c^	30	1.78	1.18–2.69 ^b^	15.4	1.91	0.39–9.26
Work-life conflict															
30–40 h	14.3	1 ^f^		12.8	1 ^f^		13.7	1 ^f^		20.6	1 ^f^		10.6	1 ^e^	
41–50 h	31	3	2.52–3.57 ^c^	18.1	1.72	1.24–2.34 ^b^	25.5	2.15	1.69–2.74 ^c^	40.9	2.7	2.23–3.26 ^c^	15.7	1.74	0.89–3.43
51–60 h	57.8	9.11	6.59–12.79 ^c^	45	6.75	4.67–9.76 ^c^	40.7	4.18	2.99–5.85 ^c^	57.9	5.87	3.99–8.64 ^c^	30.8	6.05	1.71–21.33 ^b^

Note: % refers to the prevalence of poor job quality dimensions. ^a^ *p* < 0.05; ^b^ *p* < 0.01; ^c^ *p* < 0.001; ^d^ Wald test with *p* < 0.05; ^e^ Wald test with *p* < 0.01; ^f^ Wald test with *p* < 0.001. Odds ratios are adjusted for age and occupational category.

**Table 3:** Association of working time with poor job quality by country group among women. Adjusted odds ratios (aOR) and 95% confidence intervals (95% CI). Employees working 30–60 h a week. 5th European Working Conditions Survey, 2010.

	Continental			Anglo-Saxon			Eastern European			Southern European			Nordic		
	*N* = 3298			*N* = 1003			*N* = 2042			*N* = 1963			*N* = 481		
	%	aOR	95%CI	%	aOR	95%CI	%	aOR	95%CI	%	aOR	95%CI	%	aOR	95%CI
**Intrinsic job quality**															
High time pressure															
30–40 h	46.5	1 ^f^		42.1	1		59.2	1 ^f^		55.9	1 ^f^		39.9	1 ^d^	
41–50 h	32.2	0.52	0.40–0.67 ^c^	35.9	0.81	0.56–1.15	44.4	0.5	0.41–0.64 ^c^	49.3	0.76	0.40–0.98 ^a^	26.2	0.55	0.29–1.02
51–60 h	36.1	0.59	0.29–1.20	25	0.47	0.18–1.22	44.7	0.52	0.34–0.83 ^b^	30.4	0.33	0.18–0.57 ^c^	12.5	0.12	0.01–1.34
High physical demands															
30–40 h	48.8	1 ^e^		55.8	1		49.7	1 ^f^		51.7	1		55.6	1	
41–50 h	55.3	1.35	1.06–1.72 ^b^	53.6	0.93	0.65–1.31	63.6	1.97	1.55–2.50 ^c^	48.6	0.86	0.66–1.12	40.3	0.84	0.47–1.51
51–60 h	61.1	1.54	0.76–3.15	45.8	0.76	0.33–1.71	70.6	2.49	1.52–4.09 ^c^	69.6	1.88	1.01–3.50 ^a^	25	0.27	0.05–1.57
Low skill discretion															
30–40 h	34.6	1:00 AM		49.8	1 ^f^		41.1	1:00 AM		48.7	1		46.7	1	
41–50 h	23.8	0.67	0.51–0.89 ^b^	33.8	0.53	0.36–0.79 ^b^	43	1.07	0.86–1.37	55.4	1.33	1.02–1.75 ^a^	36.1	1.09	0.61–1.98
51–60 h	33.3	1.01	0.41–2.08	16.7	0.25	0.08–0.77 ^a^	57	1.8	1.16–2.87 ^a^	38.2	0.51	0.28–0.94 ^a^	37.5	0.91	0.20–4.18
Low autonomy															
30–40 h	42.6	1		49.8	1 ^e^		48.7	1 ^e^		42.5	1		39.9	1	
41–50 h	30.7	0.67	0.50–0.91 ^a^	30.7	0.47	0.33–0.69 ^c^	54.3	1.27	0.99–1.64	46	1.03	0.77–1.38	44.3	1.57	0.89–2.79
51–60 h	27.6	0.42	0.17–1.05	43.5	0.86	0.37–2.00	63.5	1.62	1.10–3.02 ^a^	51.2	1.43	0.74–2.76	25	0.77	0.17–3.57
Low participation															
30–40 h	44.3	1		49.8	1 ^f^		46	1		49.8	1		52.8	1	
41–50 h	38.8	0.87	0.70–1.15	28.1	0.45	0.28–0.64 ^c^	45.1	0.94	0.73–1.17	46.8	0.89	0.67–1.23	41.5	0.87	0.47–1.60
51–60 h	36.1	0.6	0.30–1.29	45.8	1.12	0.48–2.48	62.4	1.87	1.16–3.00 ^a^	38.2	0.51	0.27–0.96 ^a^	37.5	0.59	0.12–2.84
Low support															
30–40 h	46.7	1		27.7	1		38.3	1 ^f^		40.3	1		32.7	1	
41–50 h	45.4	0.96	0.75–1.23	20.1	0.72	0.47–1.10	48.1	1.46	1.17–1.82 ^c^	38.8	0.97	0.74–1.87	35.5	1.25	0.68–2.28
51–60 h	60	1.55	0.74–3.27	27.3	1.23	0.48–3.13	52.4	1.74	1.11–2.72 ^a^	31.4	0.67	0.36–1.26	37.5	1.75	0.42–7.26
**Prospects**															
Non-permanent contract															
30–40 h	11.6	1 ^e^		12.5	1		16	1 ^d^		22.1	1 ^f^		10.4	1	
41–50 h	13.4	1.37	0.96–1.95	14.4	1.29	0.78–2.12	19.3	1.09	0.81–1.45	35.5	1.57	1.18–2.08 ^c^	12.9	1.29	0.59–2.82
51–60 h	25	2.16	0.93–5.00	16.7	1.49	0.49–4.53	29.4	2.05	1.23–3.42 ^b^	53.6	3.88	2.16–7.00 ^c^	12.5	2.04	0.39–10.81
Low employer-funded training															
30–40 h	46.7	1		36	1		51.1	1		64	1		29.6	1	
41–50 h	46.9	1.06	0.83–1.35	28.8	0.8	0.54–1.13	51.8	1	0.80–1.25	60.1	0.81	0.62–1.06	25.8	1.08	0.57–2.04
51–60 h	34.3	0.52	0.25–1.18	62.5	3.3	1.42–7.62 ^b^	64	1.63	1.03–2.59 ^a^	60	0.73	0.41–1.32	25	1.17	0.25–5.52
**Rewards**															
30–40 h	50.3	1		40.1	1		41.1	1 ^e^		37.6	1		40.4	1	
41–50 h	47.2	0.88	0.70–1.12	49	1.4	1.02–2.06 ^a^	35.4	0.8	0.63–1.01	46.3	1.43	1.10–1.85 ^a^	54.1	1.44	0.81–2.55
51–60 h	44.4	0.85	0.43–1.69	41.7	1	0.47–2.48	29.4	0.65	0.40–1.05	35.7	0.96	0.54–1.70	62.5	2.37	0.55–10.22
**Working-time quality**															
Changing schedules															
30–40 h	25.5	1		14.6	1 ^f^		16.7	1 ^f^		16.2	1		15.8	1	
41–50 h	29.4	1.23	0.95–1.59	26.8	2.43	1.59–3.71 ^c^	27.5	1.78	1.38–2.30 ^c^	19.4	1.17	0.84–1.63	9.8	0.65	0.27–1.58
51–60 h	22.2	0.69	0.29–1.61	33.3	3.28	1.32–8.15 ^b^	23.5	1.47	0.88–2.47	21.4	1.7	0.88–3.29	12.5	1.04	0.15–6.97
Work-life conflict															
30–40 h	16.9	1 ^f^		8.1	1 ^f^		11.9	1 ^f^		22.2	1 ^f^		10.4	1	
41–50 h	33.1	2.76	2.13–3.57 ^c^	23.4	3.6	2.28–5.71 ^c^	21.6	2.08	1.57–2.76 ^c^	33.8	1.98	1.46–2.55 ^c^	14.5	1.76	0.77–4.05
51–60 h	45.7	4.02	2.02–8.08 ^c^	29.2	4.41	1.72–11.26 ^b^	48.2	6.98	4.41–11.07 ^c^	40	2.82	1.61–4.92 ^b^	25	4.14	0.86–19.95

Note: % refers to the prevalence of poor job quality dimensions. ^a^ *p* < 0.05; ^b^ *p* < 0.01; ^c^ *p* < 0.001; ^d^ Wald test with *p* < 0.05; ^e^ Wald test with *p* < 0.01; ^f^ Wald test with *p* < 0.001. Odds ratios are adjusted for age and occupational category.
